# Dementia wellbeing and COVID‐19: Review and expert consensus on current research and knowledge gaps

**DOI:** 10.1002/gps.5567

**Published:** 2021-05-27

**Authors:** Kathy Y. Liu, Robert Howard, Sube Banerjee, Adelina Comas‐Herrera, Joanne Goddard, Martin Knapp, Gill Livingston, Jill Manthorpe, John T. O'Brien, Ross W. Paterson, Louise Robinson, Martin Rossor, James B. Rowe, David J. Sharp, Andrew Sommerlad, Aida Suárez‐González, Alistair Burns

**Affiliations:** ^1^ Division of Psychiatry University College London London UK; ^2^ Faculty of Health University of Plymouth Plymouth UK; ^3^ Department of Health Policy London School of Economics and Political Science Care Policy and Evaluation Centre London UK; ^4^ Economic and Social Research Council UK Research and Innovation Swindon UK; ^5^ NIHR Policy Research Unit in Health and Social Care Workforce King's College London London UK; ^6^ Department of Psychiatry University of Cambridge School of Clinical Medicine Cambridge UK; ^7^ Dementia Research Centre Queen Square UCL Institute of Neurology University College London London UK; ^8^ Population Health Sciences Institute Faculty of Medical Sciences Newcastle University Newcastle UK; ^9^ Medical Research Council Cognition and Brain Sciences Unit University of Cambridge Cambridge UK; ^10^ Department of Clinical Neurosciences University of Cambridge Cambridge UK; ^11^ Department of Brain Sciences Imperial College London London UK; ^12^ UK Dementia Research Institute Care Research and Technology Centre, Imperial College London London UK; ^13^ Division of Neuroscience and Experimental Psychology The University of Manchester Manchester UK

**Keywords:** COVID‐19, dementia, research, wellbeing

## Abstract

**Objectives:**

In response to a commissioned research update on dementia during the COVID‐19 pandemic, a UK‐based working group, comprising dementia researchers from a range of fields and disciplines, aimed to describe the impact of the pandemic on dementia wellbeing and identify priorities for future research.

**Methods:**

We supplemented a rapid literature search (including unpublished, non‐peer reviewed and ongoing studies/reports) on dementia wellbeing in the context of COVID‐19 with expert group members' consensus about future research needs. From this we generated potential research questions the group judged to be relevant that were not covered by the existing literature.

**Results:**

Themes emerged from 141 studies within the six domains of the NHS England COVID‐19 Dementia Wellbeing Pathway: Preventing Well, Diagnosing Well, Treating Well, Supporting Well, Living Well and Dying Well. We describe current research findings and knowledge gaps relating to the impact on people affected by dementia (individuals with a diagnosis, their carers and social contacts, health and social care practitioners and volunteers), services, research activities and organisations. Broad themes included the potential benefits and risks of new models of working including remote healthcare, the need for population‐representative longitudinal studies to monitor longer‐term impacts, and the importance of reporting dementia‐related findings within broader health and care studies.

**Conclusions:**

The COVID‐19 pandemic has had a disproportionately negative impact on people affected by dementia. Researchers and funding organisations have responded rapidly to try to understand the impacts. Future research should highlight and resolve outstanding questions to develop evidence‐based measures to improve the quality of life of people affected by dementia.

## Key Points

- The COVID‐19 pandemic has had a disproportionately negative impact on people affected by dementia, dementia services and research.
- Further research on the impacts of the pandemic is needed, and directions for future work are identified in this study.
- It is important to explore the potential benefits and risks of remote health and care, design inclusive and longitudinal studies to monitor longer‐term impacts, and report dementia‐specific findings within broader studies.

## 1 INTRODUCTION

The COVID‐19 coronavirus pandemic disproportionately affects people living with dementia who have a substantially increased risk of infection and subsequent death,^1^, ^2^ accounting for 31% of all COVID‐19 related deaths.^3^ People living with dementia are also at risk of and affected by the social isolation and reduced access to health and social care that accompany pandemic restrictions. In the UK, people affected by dementia (individuals with dementia, their carers and social contacts, health and social care practitioners and volunteers), were ‘worst hit’ by the pandemic.^4^, ^5^ Dementia services, research activities and funding have been reduced, while health inequalities have widened.^6^ Initiatives to investigate and address these impacts have begun to identify learning from pandemic experiences, but research that includes people living with dementia has been affected because of their vulnerability to COVID‐19.

For those working to support people living with dementia and carers, it is important to understand findings from existing research on COVID‐19 and dementia, and how to make the best use of that research to inform policy and practice and identify key research gaps to direct future work. In response to the impact of the pandemic on dementia wellbeing, NHS England published a guide to delivering policy aspirations in the context of COVID‐19, based on the NHS England Dementia Well Pathway for delivering best dementia care^7^. This pathway provides a framework of care and support that people with dementia, and their carers, need at each stage of their journey, from prevention to end‐of‐life,^8^ so that dementia care, research and awareness can be optimised.^9^ The guide to dementia wellbeing in the COVID‐19 pandemic^7^ highlighted priorities and actions and provided guidance and resources within six phases of dementia wellbeing: Preventing Well, Diagnosing Well, Treating Well, Supporting Well, Living Well and Dying Well. The English Department of Health and Social Care (DHSC) Dementia Programme Board subsequently commissioned a research update on dementia during the COVID‐19 pandemic.

This paper comes from a literature search for studies about dementia and COVID‐19 and the expert consensus of a working group formed in response to the question from the Dementia Programme Board in November 2020. Using the COVID‐19 Dementia Wellbeing Pathway^7^ as a framework, we aim to describe completed and ongoing research on dementia wellbeing and COVID‐19 and identify key directions for future work.

## 2 METHODS

Relevant questions, research databases and completed and ongoing studies were identified by members of the working group during an initial meeting and subsequent correspondence. Expertise of the UK‐based working group included researchers from health, economic and social care who work on dementia‐related topics (see Table S1 for further details of the working group). Initial discussions were supplemented by a literature search to identify published and unpublished research on COVID‐19 and dementia at the end of 2020. We identified areas of consensus on the important gaps in the literature and future directions for research. We report findings aligned with the six steps of the COVID‐19 Dementia Wellbeing Pathway and specifically mention the UK studies.

### 2.1 Literature search and study selection

One author (KL) led the literature search, reviewed titles and abstracts for relevance and then extracted data from relevant full texts. Databases were searched using specific search terms (Table 1) on 9th December 2020, which was updated on 7th January 2021 in consultation with members of the expert group.

**TABLE 1:** Databases and search terms used for the literature search

Database type	Database name	Search terms
Published studies	PubMed	‘(SARS‐CoV‐2 OR COVID‐19 OR hCoV‐19 OR 2019‐nCoV) AND (dementia OR Alzheimer OR neurodegenerative OR “mild cognitive impairment”)’ in titles, abstracts and full texts.
	Web of Science	
Registered systematic reviews	PROSPERO	
Preprint databases	BioRxiv and MedRxiv (these did not support parentheses for the search)	‘Covid AND dementia’ within titles and abstracts
Ongoing UK funded studies	Long Term Care (LTC) Responses to COVID‐19 project^10^	Projects related to ‘dementia’
	COVID‐19 UK Research and Innovation (UKRI) grants databases^11^	
	UK National Institute for Health Research (NIHR) Funding and Awards database^12^	Projects related to ‘dementia’ and ‘COVID’

Included studies were research articles in the English language from any country on COVID‐19 and dementia or mild cognitive impairment in individuals of any age. Individuals with mild cognitive impairment are often assessed and/or followed up by memory services, and a significant proportion are experiencing the early stages of a neurodegenerative illness and will progress to dementia. We included primary studies and reviews, as well as case series, commentaries, position papers and policy documents if they reported quantitative or qualitative findings in relation to COVID‐19 and dementia wellbeing. We excluded studies that investigated risk for development of dementia secondary to COVID‐19, the mechanisms of disease, or the impact of dementia on COVID‐19 mortality or infection, without identifying specific risk factors within dementia. Case reports were excluded, as studies of individuals were judged to be insufficiently informative for the purpose of this study, as were articles based purely on data that were not obtained during the COVID‐19 pandemic period (before November 2019).

**FIGURE 1:**
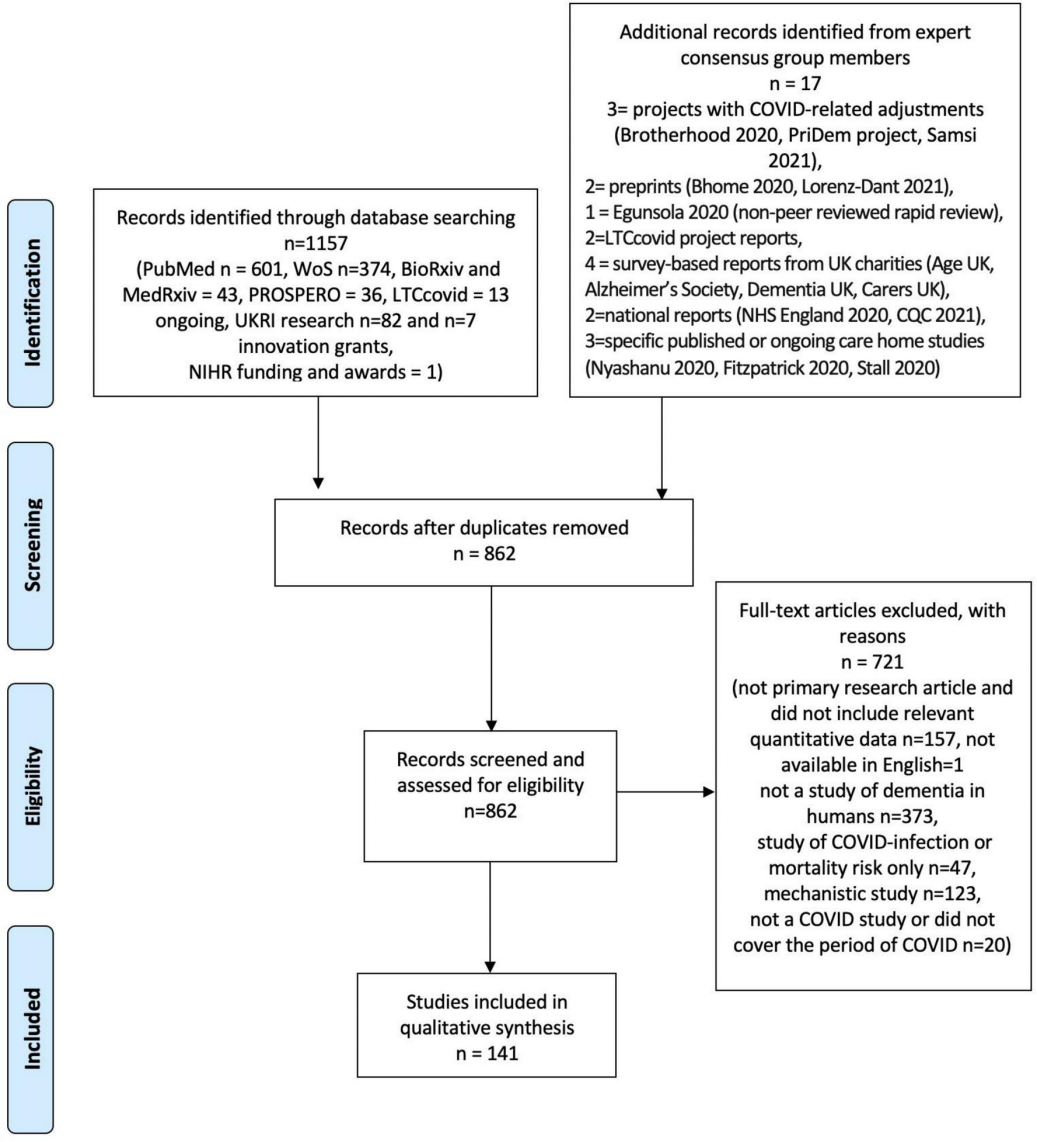
The number of records identified, included and excluded, and the reasons for exclusions. *Adapted from*: Moher D, Liberati A, Tetzlaff J, Altman DG, The PRISMA Group (2009)^13^

Any additional studies or reports that the expert group judged to be consistent with the aim of identifying the scope of existing research on COVID‐19 and dementia wellbeing, but that had not emerged from the literature search, were combined with the search results and reported (Figure 1). For example, some specific studies on people living or working in long‐term care facilities, such as care homes, did not explicitly report that participants with dementia were included in the study, but were judged to be relevant as more than 70% of UK care home residents are estimated to have dementia.^14^, ^15^ Some expert members were also aware of ongoing funded studies that had adjusted their existing project to explore the impact of COVID‐19, non‐peer reviewed UK survey‐based reports from charities,^4^, ^5^, ^16^, ^17^ as well as two reports published by the ‘LTCcovid’ collaboration (www.ltccovid.org), which was set up to gather resources to support long term care responses to COVID‐19.^3^, ^18^ Some members were aware of funded studies that have yet to be announced; we have not included them in the tables but provide some details in the main text.

## 3 RESULTS

**FIGURE 2:**
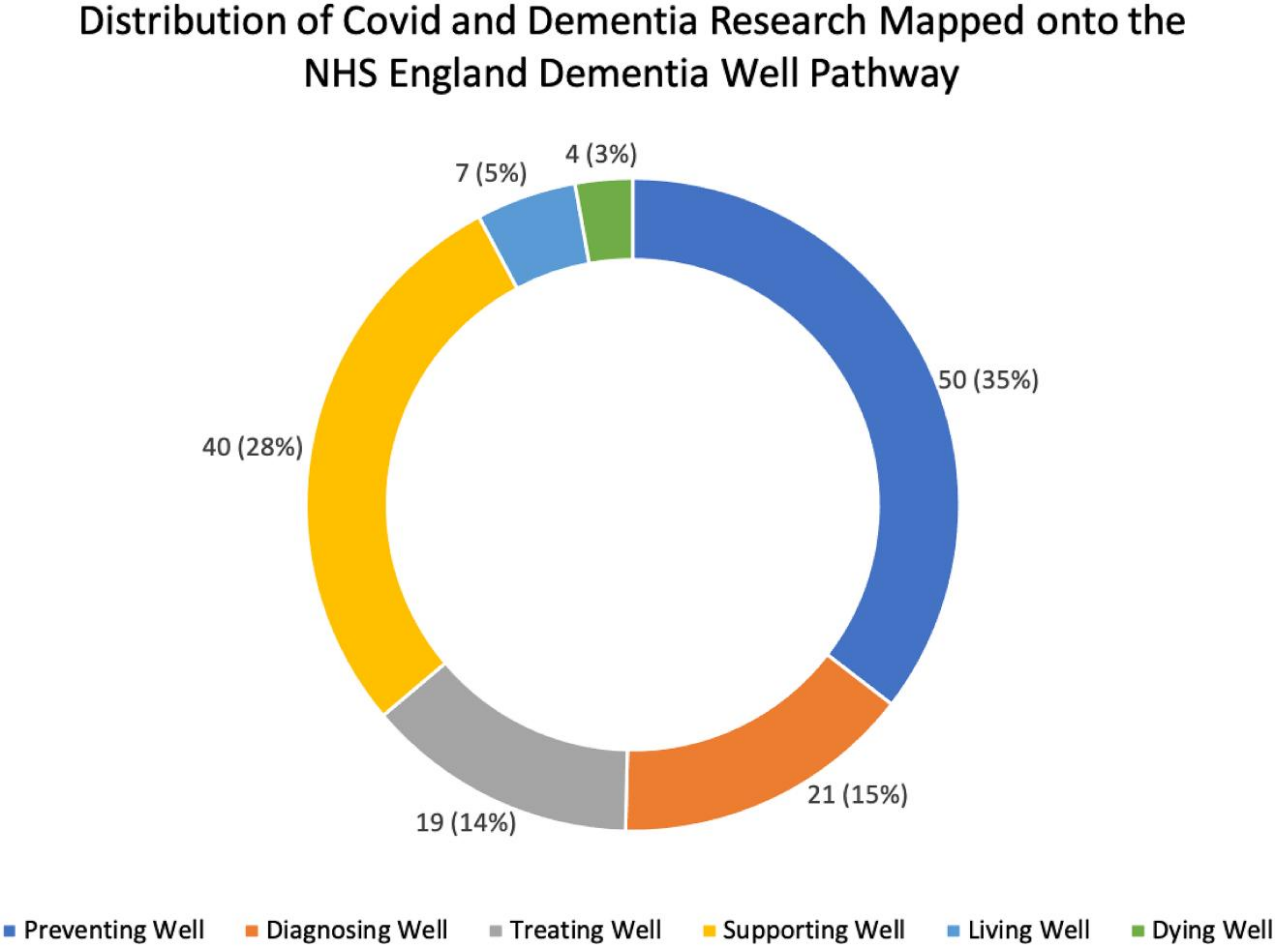
Distribution of COVID‐19 and dementia wellbeing research mapped onto the NHS England Dementia Well Pathway. Figures show number of studies (percentage of total)

The database searches and screening of articles identified 141 relevant studies (see Figure 1, 2 and Tables 2, 3, 4, 5, 6, 7). We describe the main findings from these studies and key gaps in research identified by expert consensus in relation to each of the six steps of the COVID‐19 Dementia Wellbeing Pathway, summarised in Box 1.

**TABLE 2:** Preventing well

Study	Type	Country	Study type/methodology	Number of participants (with dementia if applicable)	Type of dementia	Setting	Focus of study	Findings	Themes
Maurik 2020^19^	1	Netherlands	Survey of cohort	389 (121)	AD, DLB, MCI	Home‐dwelling	Psychosocial effects of pandemic restrictions on dementia/MCI patients, their caregivers and patients with subjective cognitive decline.	Significant proportion of patients experienced higher rates of social isolation and behavioural problems, which were associated with higher carer burden.	Impact of pandemic restrictions on behaviour, activity and carer wellbeing.
Palermo 2020^20^	1	Italy	Prospective cohort	28	PD, MCI	Home‐dwelling	Effects of pandemic restrictions on patients.	Worse anxiety and cognition, no change in depression, sleep or motor symptoms.	
Di Santo 2020^21^	1	Italy	Telephone interview	126 (70)	MCI	Home‐dwelling	COVID‐19 restriction impact on wellbeing.	Reduction in productive leisure activities, which was associated with anxiety symptoms.	
Manca 2020^22^	1, 3	NA	Review	NA	NA	NA	Impact of infection or social isolation due to COVID‐19 on older adults with or without dementia.	COVID‐19 has a wide negative impact on wellbeing in older adults with dementia and without.	
Tsapanou 2020^23^	1	Greece	Cross sectional survey	204 dyads	MCI and dementia	Home‐dwelling	Study the impact of the COVID‐19 pandemic on mental and physical effects on people with MCI/dementia and their carers.	Overall decline in mood, communication, movement and compliance with new measures in patients. Carers experienced greater burden.	
Lara 2020^24^	1	Spain	Prospective cohort	40 dyads	MCI, AD	Home‐dwelling	Impact of pandemic on neuropsychiatric symptoms and quality of life 1 month before and 5 weeks after pandemic lockdown.	Neuropsychiatric symptom burden increased, most frequently agitation, apathy, aberrant motor activity. No difference in quality of life scores.	
Goodman‐Casanova 2020^25^	1	Spain	Cross‐sectional survey	93	MCI or mild dementia	Home‐dwelling	Study the impact of COVID‐related confinement on wellbeing of participants on an existing study looking at TV‐assisted support.	Most participants had good wellbeing, but those living alone reported greater negative psychological and sleep issues. No difference between study groups. Those in the intervention arm performed more memory exercises on TV.	
O'Caoimh 2020^26^	1	Ireland	Cross sectional online survey	202 (161) dyads	‘Cognitive impairment’	Residential care facilities	Visitors of residents were surveyed to assess effects of COVID‐19 on wellbeing.	Visitors experienced low psychosocial and emotional wellbeing during lockdown, and visitors of residents with cognitive impairment had worse well being than those without.	
Nyashanu 2020^27^	1	UK	Semi‐structured interview	40 healthcare workers	NR	Care homes	Explored triggers of mental health problems in healthcare workers.	Triggers included fear of infection and infecting others, lack of recognition/disparity between NHS and social care, lack of guidance, unsafe hospital discharge, death and loss of professionals and residents, unreliable testing and delayed results and shortage of PPE.	
Cohen 2020^28^	1	Argentina	Survey cross‐sectional	119 dyads	AD and mixed.	Home‐dwelling	Family caregivers were asked to report change in behavioural symptoms after the first 8 weeks of mandatory quarantine.	There was an overall deterioration behaviour in patients, probably related to the social restrictions and lack of outpatient services. Carers experienced increased stress.	
Cagnin 2020^29^	1	Italy	Survey cross‐sectional	4913	AD, FTD, DLB, VD	Home‐dwelling	Carer views of neuropsychiatric symptoms 1 month after pandemic restrictions introduced.	There was an increase in neuropsychiatric symptoms in over half of patients and stress related symptoms in two thirds of carers. AD was associated with anxiety and depression, DLB with worsening hallucinations and sleep disorder, FTD with wandering and change of appetite.	
Boutoleau 2020^30^	1	France	Cross‐sectional survey	38 dyads	AD	Home‐dwelling	Carer views of neuropsychiatric symptom change during COVID restrictions	26% of patients had a change in neuropsychiatric symptoms. They had lower cognitive function compared to others and length of confinement correlated with symptom severity and carer stress.	
El Haj 2020^31^	1	France	Prospective cohort	58	AD	Retirement homes	Change in depression and anxiety in retirement homes during and before the COVID pandemic.	Higher depressive and anxiety symptoms during compared to before the pandemic. This was attributed to social restrictions in the homes.	
Carpinelli Mazzi 2020^32^	1	Italy	Cross sectional	239 dyads	AD, VD, mixed, DLB	Home‐dwelling	Psychological impact of COVID lockdown on carers	Lockdown had negative effects on half of patients. Higher levels of anxiety and depression with time of isolation. Higher education was a protective factor and being female was a risk factor.	
Altieri 2020^33^	1	Italy	Cross section survey	84 dyads	AD, VD, FTD	Home‐dwelling	Psychological impact of COVID lockdown on carers, and levels of resilience	Carers experienced increased depression. High resilience was associated with lower anxiety and caregiver burden but not related to depressive symptoms.	
Alexopoulos 2020^34^	1	Greece	Cross sectional	67 dyads	AD, VD, mixed, FTD	Home‐dwelling	The relationship between caregiver stress and neurocognitive symptoms in the people they care for.	Caregiver distress severity was related to memory deficits, neuropsychiatric symptoms, caregiver hyperarousal, avoidance and COVID‐related worries.	
Borges‐Machado 2020^35^	1	Portugal	Prospective (pre and post)	36 dyads	Dementia or neurocognitive disorder	Home‐dwelling	Compare pre‐ and post‐ impact of pandemic (Nov 2019 and Jun 2020) on impact by surveying carers.	Care recipients had decreased independence and increased NPI score. Caregivers reported decline in wellbeing and increased burden.	
Penteado 2020^36^	1	Brazil	Cross sectional	100 (74)	MCI, AD,VD, FTD	Home‐dwelling	Survey and interview of older adults and aging adults with Down syndrome with neurocognitive disorders.	Neuropsychiatric symptoms and caregiver burden were higher in people with dementia.	
Soldevilla 2020^37^	1	Spain	Prospective	16	At risk of AD (subjective cognitive decline and APOE4 carriers)	Home‐dwelling	Follow up assessments to assess mental and cognitive health during the pandemic lockdown.	Mood deteriorated during and after lockdown but cognition remained stable.	
Roach 2020^38^	1	Canada	Remote interview	21 dyads	NR	Home‐dwelling	Assess impact of pandemic on carers and people with dementia.	Isolation and mental health needs were identified as important.	
Thyrian 2020^39^	2	Germany	Telephone interview	141	Mild dementia	Home‐dwelling	Assess impact of pandemic on people with cognitive impairment who were already enrolled in mild cognitive impairment or dementia trials.	Reported limited impact of the pandemic and identified a need for longitudinal studies.	
Baschi 2020^40^	1	Italy	Cross‐sectional	96 (62) dyads	PD‐MCI, MCI	Home‐dwelling	Assess impact of pandemic on prodromal PD dementia through a post‐lockdown interview	Reported worsening of cognitive, behavioural and motor symptoms, especially in PD‐MCI.	
Koh 2020^41^	1	Singapore	Retrospective	634	NR	Home‐dwelling	Comparison of records from patients who were assessed by a geriatric clinic pre‐ and during pandemic lockdown.	Higher behavioural symptoms in group during lockdown versus group pre‐lockdown.	
Barguilla 2020^42^	1	Spain	Prospective	60 dyads	AD, MCI, VD, FTD, DLB	Home‐dwelling	Impact of pandemic by comparing post‐ and pre‐lockdown data.	Reduction in social activities, increase in carer burnout and burden, worsening of dementia‐related behaviours reported.	
Iodice 2021^43^	1, 3	NA	Review	NA	NA	NR	Review of impact of pandemic on older people including with cognitive impairment such as dementia.	There has been reduced support to older people with dementia during the pandemic which needs to be addressed.	
Suarez‐Gonzalez 2020^3^	1	UK	Cross sectional survey	184 carers and 84 people with dementia	Rare and young‐onset dementia	Home‐dwelling	Assess impact of COVID pandemic	Worsening of dementia symptoms and reduced support were reported.	
Jeon 2020^44^	5	Australia	Cross sectional survey	NR (dyads)	NR	NR	Examine the impact of COVID‐19 on life and wellbeing and access to care and support of people living with dementia and their care partners, and identify key issues and lessons.	Interim report published, ongoing.	
Porcari 2020^45^	1	Italy	Observational/cross‐sectional	150 dyads	NR	Home‐dwelling	Development of 2 surveys (caregiver or patient) to be used to monitor the impact of the pandemic. Ongoing study.	NA	
Whitley C19‐IUC‐382 (UKRI)	5	UK	Survey	NR	NR	NR	The impact of COVID restrictions on the mental health of carers	NA	
Giebel 2021	5	UK, Poland, Italy, Australia, India	Mixed methods	NR	NR	NR	International impact of COVID restrictions on unpaid carers and people with dementia.	NA	
Giebel 2021	5	UK	Qualitative interview	40	NR	NR	Experiences of care home staff and family carers of people with dementia residing in a care home during the pandemic.	NA	
Daley 2021^46^	5	UK	Mixed methods	350 dyads	NR	NR	To understand how COVID‐19 has affected the quality of life, wellbeing and care of people with dementia and their carers, compared to pre‐COVID measures.	NA	
Suarez‐Gonzalez et al., 2021^47^	5	NA	Rapid review	NA	NR	NR	Rapid review of impact of pandemic and isolation of people with dementia.	NA	
Stockwell 2021^48^	3, 5	NA	Registered systematic review	NA	NR	NA	To review the prevalence of physical and sedentary behaviours during the pandemic, to include people with dementia.	NA	
Alzheimer's Society UK^4^	6	UK	Survey, analysis of existing records and publications	128 care home managers, 134 people with dementia, 1000 carers and available records on 2000 people.	NR	Included home‐dwelling and care homes	Report on the impact of the pandemic on people affected by dementia.	Half of people with dementia who were surveyed reported worse mental health, 82% of carers surveyed reported deterioration in dementia symptoms, 95% carers reported worse mental health, 75% care homes managers reported that GPs were reluctant to visit in May 2020%, 93% of users reported they were more able to manage after support provided by Alzheimer's Society.	
Dementia UK^5^	6	UK	Survey	169 carers	NR	NR	Report included the impact of pandemic on carers.	83% carers said they had fewer opportunities to take a break from caring, 78% found it harder to cope and had negative impact on wellbeing of the person they care for, 86% reported negative impact on own wellbeing. 71% said home visits from care staff were cancelled, 57% were offered alternative e.g. telephone appointments, half those offered telephone appointments said that this met their needs or those of the person they care for. Many were pessimistic about future support.	
Age UK^16^	6	UK	Report using online survey/polling.	NR	NR	NR	Report on impact of pandemic on older people that included a section on older people with dementia.	Family members reported that the people with dementia they cared for deteriorated in their behaviour related to the pandemic.	
Carers UK^17^	6	UK	Online survey	4830 carers and 217 former carers	NR	NR	Report on impact of pandemic on carers. 22% of those surveyed were aged >65 years.	70% were providing more care due to the pandemic, 35% were doing this due to local services closing, 50% felt overwhelmed and worried about burnout.	
								See also Lorenz‐Dant 2021^49^ (Table 4)	
Kobayashi 2020^50^	1	Japan	Semi‐structured interview	55 dyads	AD	Home‐dwelling	Understanding of why face masks need to be worn and ability to wear them.	Most AD patients were unable to explain why they needed to wear a face mask and most could not put one on independently. A proportion could not do so even with help from a carer.	Reduced ability to understand and comply with pandemic restrictions.
Suzuki 2020^51^	1	Japan	Qualitative, semi‐structured interviews	24 dyads	AD, FTD	Home‐dwelling	How FTD and AD patients and their carers managed to comply with restrictions.	FTD patients showed more difficulty keeping social distances, washing hands and staying at home, compared to AD.	
Tsugawa^52^	1	Japan	Qualitative semi‐structured interviews	126	AD	Home‐dwelling	Awareness of the COVID‐19 pandemic and need to wear masks, May‐Jun 2020.	Moderate‐severe AD patients were less likely to be aware of the pandemic or understand the need to wear masks, compared to mild AD. They were also more likely to have depressive symptoms.	
Hashimoto 2020^53^	1	Japan	Questionnaire	111 (74)	AD, FTD, DLB, MCI	Home‐dwelling	Lifestyle changes due to COVID‐19 during Apr 2020.	Most MCI and dementia patients who lived alone did not limit their outings or activities during one month of the outbreak.	
Kuo 2020^54^	1	UK	Prospective cohort	322948 (1090)	NR	Home‐dwelling	APOE‐4 genotype and COVID‐19 risk.	APOE‐4 homozygosity increased risk of severe COVID‐19 infection in dementia and non‐dementia participants.	Specific COVID19 risk factors in people with dementia.
Sainz‐Amo 2020^55^	1	Spain	Case‐control	211 (38)	PD	Hospital	COVID‐19 risk in PD.	Institutionalization and presence of neoplasm increased risk and severity of COVID‐19, and not PD‐related variables.	
De Smet 2020^56^	2	Belgium	Retrospective observational	81 (36)	NR	Hospital	Association between frailty and COVID mortality in hospitalized patients.	Mortality was not associated with dementia, but dementia was associated with frailty which was related to COVID mortality.	
Li 2021^57^	3, 5	NA	Registered systematic review	NA	NR	NA	Review of frailty risk on COVID mortality, to include dementia as subgroup analysis.	NA	
Wardlaw C19‐IUC‐044 (UKRI)	5	UK	NR	NR	VD	NR	Assess impact of COVID‐19 on stroke patients with vascular disease who are in an existing vascular dementia follow up study.	NA	
Dutey‐Magni 2020^58^	2	UK	Prospective cohort	8713 (3419)	NR	LTC facilities	SARS‐CoV‐2 infection and mortality in LTC facilities, Mar‐Jun 2020.	20% had symptoms during pandemic but few were tested. Lower staffing ratios and higher occupancy rates were independent predictors of infection.	Crowding, staffing and other LTC facility‐related factors.
Brown 2020^59^	1	Canada	Prospective cohort	78607 (54868)	NR	Nursing homes	The impact of crowding (number of residents per bathroom and bedroom) on COVID‐19 infection and mortality.	Crowding was common in nursing homes and associated with larger and deadlier COVID outbreaks.	
Frazer 2020^60^	2, 3	NA	Systematic review. Dementia mentioned in one source (Office of National Statistics, UK).	NA	NA	NA	Assess impact of various prevention strategies on COVID‐19 infection in LTC facilities.	Mass testing was the primary prevention measure used. No distinct patterns between prevention strategies and infection prevalence. Factors such as larger facility size, more staff, staff who worked across multiple facilities, higher number of infected staff, no sick leave, reduced availability of PPE, for‐profit status, were more likely to result in COVID‐19 outbreak.	

*Note:* Type: 1 = published article, 2 = preprint, 3 = review, 4 = study protocol, 5 = (funded) study to be completed, 6 = national or international report.

Abbreviations: AD, Alzheimer's disease; DLB, Lewy body dementia; FTD, frontotemporal dementia; LTC, long term care; MCI, mild cognitive impairment; MDT, multidisciplinary; NA, not applicable; NR, not reported; PD, Parkinson's disease; PPE, personal protective equipment.

**TABLE 3:** Diagnosing well

Study	Type	Country	Type of study	Number of participants (with dementia if applicable)	Type of dementia	Setting	Focus of study	Findings	Themes
Nizama‐Via 2020^61^	1	UK	Cohort retrospective	17 (6)	NR	Psychiatric hospital	Characterization of inpatients on old age ward.	All patients with dementia presented with symptoms, a quarter of whom had hypoactive delirium.	Presentations of COVID‐19 and testing.
Bianchetti 2020^62^	1	Italy	Retrospective cohort	82	NR	Acute hospital	Assess prevalence, clinical presentation and outcomes of dementia patients with COVID.	Dementia, especially advanced dementia, is a risk factor for COVID mortality. The most frequent symptom of onset was delirium, especially hypoactive delirium, and worse functional status.	
Louie 2020^63^	1	USA	Retrospective cohort	21 (15)	NR	LTC facility	Clinical characteristics of first COVID‐related fatalities in LTC residents in San Francisco (LTC and non‐LTC).	Nearly half of LTC residents had atypical symptoms, without fever, cough or dyspnoea. In a quarter, altered mental status was the only symptom.	
Rebora 2020^64^	1	Italy	Observational cohort	516 (85)	NR	Hospital	Assess relationship between delirium and COVID mortality, and risk factors for delirium.	Dementia was a risk factor for delirium in hospitalised patients, which in turn increased risk of COVID mortality.	
Canevelli 2020^65^	1	Italy		415	NR	Hospital	Clinical characteristics of dementia patients who died from COVID between Feb‐Apr 2020.	Dementia patients were less likely to present with cough, to have faster clinical deterioration, have reduced access to intensive care, antivirals, hydroxychloroquine and chloroquine.	
Rutten 2020^66^	1	Netherlands	Prospective cohort	4007 (2364)	NR	Nursing home	Symptomatology and mortality risk due to COVID‐19.	Overlap in symptomatology between COVID positive and negative residents. Higher mortality in residents with dementia.	
Livingston 2020^67^	1	UK	Retrospective cohort	131 (74)	Late and young‐onset dementia	Psychiatric hospital	Prevalence, management and outcomes of COVID‐19 in older adult mental health wards.	Patients had a higher risk of infection and mortality compared to those in the community. COVID‐testing was not available in the initial stages of the pandemic.	
Graham 2020^68^	1	UK	Prospective cohort – Two point prevalence surveys	394 (223)	NR	4 nursing homes	SARS‐CoV‐2 infection, clinical features and outcome.	40% COVID‐positive testing residents were asymptomatic and 18% had atypical symptoms only. Some asymptomatic staff also tested positive. 26% of residents died during the two months of the study.	
Graham 2020^68^	1	UK	Cross‐sectional	241 (NR)	NR	4 nursing homes	SARS‐CoV‐2 seroprevalence in the same four nursing homes as above.	72% of nursing home residents were antibody positive. 93% of previous COVID‐test positive residents had developed detectable antibodies.	
Pranata 2021^69^	3, 5	NA	Registered systematic review	NA	NR	NA	Systematic review of delirium on COVID mortality risk, including whether this is associated with comorbidities including dementia.	NA	
Weiss 2020^70^	1	USA	Experience of one centre	85	NR	Home‐dwelling	Description and lessons from a telecare program in response to COVID Mar‐May 2020.	Most patients attended appointments via the telecare program and support and advice could be given to patients and carers.	Remote assessment and virtual diagnosis of dementia.
Capozza 2020^71^	1	Italy	Cross sectional	32	FTD	Home‐dwelling	MDT assessment of patients using telehealth in Apr 2020.	Most patients were satisfied with telehealth interview. They detected significant worsening in behaviour and language in half the patients since the start of lockdown.	
Owens 2020^72^	1, 3	NA	Literature review and experience of authors (UK)	NA	NA	NA	Present a framework for virtual memory clinic assessment.	A potential pathway with 3 levels of complexity described: 1) telephone/video based interviewing and using available tests, 2) digitized and validated tests based on pen‐and‐paper tests, 3) fully digitized test batteries and remote measurement technologies.	
Geddes 2020^73^	1, 3	USA	Review	NA	NA	NA	Search for studies published between 2000 to Jun 2020 on remote cognitive assessment, integrated with MDT expert opinions.	Telemedicine has potential to provide remote cognitive assessment during the pandemic and beyond. It will be important to ensure that reliance on technology does not marginalize sections of the population.	
Carlew 2020^74^	1, 3	NA	Review	NA	NA	NA	Review of telephone based cognitive assessments up to Apr 2020.	Included studies that investigated cognitive assessments for dementia. Telephone‐based assessments are viable, and while not intended to replace face‐to‐face, can expand scope of practice.	
Domingues 2020^75^	1, 3	NA	Review	NA	NA	NA	Review of telemedicine use in neurology, including for dementia, up to Jun 2020.	Telemedicine can be useful and viable for people with dementia.	
NHS England^76^	1, 6	UK	Report	NA	NA	NA	Guidance for Memory Assessment Services in UK in light of the pandemic, including a discussion on virtual assessments.	Three core principles included that services should be needs led, there should be equality of access, and risks should be assessed and monitored.	
Sharp and Barnaghi C19‐IUC‐032 (UKRI)	5	UK	NR	100 dyads	NR	Home‐dwelling	A new protocol to an existing remote technology programme to monitor for symptoms of COVID‐19.	NA	
Michalowsky 2020^77^	1	Germany	Cross‐sectional	2.45 million older adults (healthy)	NA	Mixed	Compared the number of consultations, referrals, admissions, and incident diagnoses during lockdown in 2020 to 2019.	38% reduction in dementia diagnoses in the 2020 lockdown period compared to the previous year.	Dementia diagnosis rates and secondary care referrals
Chen 2020^78^	1	UK	Interrupted time series study	∼0.86 million	NR	Community	Assess the medium‐term impact of COVID on referrals to secondary care mental health services	No post‐lockdown longer‐term acceleration rate for referrals of people with dementia, compared to an overall increase for mental health referrals.	
Spalletta 2020^79^	1	Italy	Experience of one centre	Variable	NR	Community	Compared cancellation rates for first or follow up appointments for memory services during pandemic compared to previous year.	66.7% and 77.4% of patients missed their first and follow up appointments to memory service respectively during the pandemic due to pandemic restrictions.	
								See also Dementia UK and Alzheimer's Society UK reports.	

*Note:* Type: 1 = published article, 2 = preprint, 3 = review, 4 = study protocol, 5 = (funded) study to be completed, 6 = national or international report.

Abbreviations: AD, Alzheimer's disease; DLB, Lewy body dementia; FTD, frontotemporal dementia; LTC, long term care; MCI, mild cognitive impairment; MDT, multidisciplinary; NA, not applicable; NR, not reported; PD, Parkinson's disease; PPE, personal protective equipment.

**TABLE 4:** Treating well

Study	Type	Country	Type of study	Number of participants (with dementia if applicable)	Type of dementia	Setting	Focus of study	Findings	Themes
Ousset 2020^80^	1	France	Experience of one centre	Variable	AD	Home‐dwelling	Impact of COVID‐19 pandemic on clinical and research activities in Mar 2020	Memory clinic activity and outpatient visits stopped after lockdown. Teleconsultation started the following week. The number of patients received at the Research centre dropped from around 52 to 5 visits per week.	Impact on services and/or research
Benaque 2020^81^	1	Spain	Experience of one centre	Variable	AD, LBD, PD, FTD, VD	Home‐dwelling	Number of weekly visits by a memory service before and after pandemic lockdown.	Weekly visits dropped by 60% following lockdown, however telemedicine adaptations meant that 78% of the visits averaged in the weeks before confinement began could be achieved.	
Schilling 2020^82^	1	USA	Experience of one centre	167 (33)	AD, PD	Home‐dwelling	Results from a 3 week period of COVID‐19 screening and adaptation to national recommendations.	The centre remained open to clinical research activities but new patient recruitment decreased. Staff attendance and patient ongoing participation were high. There was a relatively low number of patients with COVID symptoms but no tests were performed.	
Abdelnour 2020^83^	1	Spain	Experience of one centre	130 (>78)	AD	Home‐dwelling	How the centre adapted to COVID restrictions and adverse events and safety data, from Mar‐Jun 2020.	A specific protocol to manage trial adverse events and suspected COVID cases was established. Social distancing, PPE for research personnel and SARS‐CoV‐2 PCR testing for all participants and research personnel. Only 1/108 participants dropped out of any RCT.	
Di Lorito 2020^84^	1, 4	UK	RCT protocol for qualitative study	NA	NR	Home‐dwelling	Due to the pandemic, the activity and exercise from home study was changed so that the interventions will be delivered remotely.	Study will assess how the pandemic impacted the delivery of the activity and exercise programme and how these changes were experienced by patients.	
Schwab 2020^85^	1	USA	Experience of one centre	NR	AD	NR	Report on adjustments and solutions by the research group toward generating a virtual ADAS‐Cog for use in AD clinical trials.	ADAS‐Cog was completed virtually for AD research participants. The validity and reliability of this method are pending assessment.	
Abate 2020^86^	1	Italy	Case series	3 (1)	PD	Home‐dwelling	Experience of remote management via video and phone.	Remote advanced management was possible and helpful for troubleshooting in these cases.	Remote management of dementia.
Bhome 2020^87^	2	UK	Mixed methods	158 staff	NA	Home‐dwelling and mental health inpatient	Explore mental health staff perspectives on staff and patient wellbeing and identify key challenges and innovations.	For inpatient staff, a significant challenge was infection control. Community staff identified a lack of access to physical and social care as well as reduced contact with friends and families as being challenges for patients. Remote working was seen as a positive innovation along with Covid‐19 related guidance from various sources and peer support.	
Capozzo 2020^71^	1	Italy	Survey	38 (4)	ALS‐FTD	Home‐dwelling	Feasibility of MDT assessment of ALS patients during COVID pandemic.	Telemedicine was acceptable and feasible.	
Tuijt et al., (PriDem)^88^	5	UK	Semi‐structured interviews by telephone or video call	30 patients living with dementia and 31 carers	NR	Community living	Adaptations made to the ongoing Primary care‐led post diagnostic dementia care (PriDem) project to explore the post‐diagnostic healthcare experiences of service users.	People living with dementia and their carers felt check‐up calls were reassuring but limited in scope and content. Some avoided healthcare services, wishing to minimise COVID‐19 risk, reduce NHS burden, or encountering technological barriers.	
Steare 2021^89^	3, 5	UK	Registered systematic review	NA	NR	NA	Review on remote working in mental health services in COVID, and will include studies on dementia.	NR	
Ty 2021^90^	3, 5	NA	Registered systematic review	NA	NR	NA	A review to assess whether neurology patients (including dementia) are satisfied with telehealth versus face‐to‐face.	NR	
Kerslake 2020^91^	1	UK	Case series	12	Mixed AD + VD, AD	Psychiatric hospital	Describe the clinical course in 12 patients, 9 of whom were suspected or confirmed to have COVID‐19.	They required a high level of medical input, experienced nursing care to encourage adequate food and fluid intake, and dietician input. Self reporting of COVID symptoms is unreliable. Early discussions relating to end of life/resuscitation are essential. Weight loss, dehydration and rising serum sodium secondary to COVID are worrying signs.	Hospital treatment
Li 2020^92^	1	China	Retrospective cohort	42 (19)	AD	Hospital	Clinical outcomes in hospitalized AD versus non‐AD COVID‐19 positive patients.	AD patients were hospitalized sooner and had better outcomes than non‐AD patients.	
Gomez‐RRamiro^93^	1	Spain	Retrospective analysis pre‐and post‐lockdown	1958 (11)	NR	Hospital	Differences in emergency admission rates pre versus post‐lockdown.	Overall admission rates decreased but dementia admissions increased.	
								See also Canevelli 2020 and Livingston 2020 from ‘Diagnosing well’.	
Shea 2020^94^	1	Hong Kong	Case series	3	AD	Hospital	Description of neuropsychiatric symptoms related to social isolation during the pandemic.	Changes in daily routine and social isolation were believed to underlie the worsening in behaviour in these patients. They were all prescribed antipsychotic medication.	Managing psychological and behavioural symptoms.
Simonetti 2020^95^	1, 3	NA	Systematic review	NA	NA	NA	Impact of COVID‐19 on neuropsychiatric symptoms in dementia	Apathy, anxiety and agitation were most frequently reported during COVID‐19 pandemic and appear to arise from social restrictions. Most treatment strategies rely on pharmacotherapy and there is increasing use of remote technology.	
Howard 2020^96^	1	UK	Cohort	National prevalence	NR	All	Proportion of patients with dementia prescribed antipsychotic medication.	Higher proportion of antipsychotic prescribing in dementia during COVID‐19 pandemic compared to previous years.	
Stall 2020^97^	2	Canada	Population based	77,291 residents (NR)	NR	Nursing homes	Examine the proportion of residents who were prescribed antipsychotics during the pandemic.	Increased prescribing of psychotropic drugs at the onset of COVID pandemic that persisted through Sep 2020.	

*Note:* Type: 1 = published article, 2 = preprint, 3 = review, 4 = study protocol, 5 = (funded) study to be completed, 6 = national or international report.

Abbreviations: AD, Alzheimer's disease; DLB, Lewy body dementia; FTD, frontotemporal dementia; LTC, long term care; MCI, mild cognitive impairment; MDT, multidisciplinary; NA, not applicable; NR, not reported; PD, Parkinson's disease; PPE, personal protective equipment.

**TABLE 5:** Supporting well

Study	Type	Country	Type of study	Number of participants (with dementia if applicable)	Type of dementia	Setting	Focus of study	Findings	Themes
Verbeek 2020^98^	1	Netherlands	Cross sectional, mixed methods	954 (NR)	NR	Nursing homes	Study of the impact of re‐opening nursing homes for visits after introduction of national guidelines.	Allowing visits to nursing homes after restrictions had a positive impact on wellbeing. There was variation in how the national guidelines were applied between homes.	Care home support and visiting care homes
Shum 2020^99^	1	Hong Kong	Case control	24 in 2020	AD, PD, VD mixed, other	Hospital	Report of patients admitted to hospital due to poor oral intake since the start of pandemic visitor restrictions between Jan‐May 2020.	Oral feeding may deteriorate during visitor restrictions. During the same period in 2019, there were only 8 dementia patients admitted with unexplained poor oral intake. In 2020 a higher proportion of patients received NG tube feeding, which may have been related to lack of advance care planning and visitor restrictions to hospitals.	
Lombardo 2020^100^	1	Italy	Cross sectional survey	1356 nursing homes including 100806 residents (estimated 26% had dementia)	NR	Nursing homes	Study of frequency of adverse events in nursing homes and associated factors during the pandemic.	A higher bed capacity, increased psychotropic medication use, adopting physical restraint measures, hospitalization due to flu‐like symptoms, and specific geographic areas were associated with adverse events.	
Leontjevas 2020^101^	1	Netherlands	Mixed methods	323 (>200 worked on a unit for dementia) practitioners	NR	Nursing homes	Study of nursing home practitioners' perspective of challenging behaviour during COVID‐19 pandemic.	Higher proportion of practitioners reported increased versus decreased challenging behaviors. Half reported their workload increased and work satisfaction decreased. Strategies included video calls, special meeting areas, adjusting activities reducing exposure to negative news.	
Dickson ES/P008224/1 (UKRI)	5	UK	Survey	NR	NR	Nursing home	Understand and improve antimicrobial prescribing in care homes by surveying care home staff and GPs.	NA	
Shallcross ES/V003887/1 (UKRI)	5	UK	NR	NR	NR	Nursing homes	Assess the impact of COVID on care home staff and residents, learn rapid lessons and identify pragmatic solutions.	NA	
Knight MR/V028502/1 (UKRI)	5	UK	NR	NR	NR	Care homes	Assess the impact of COVID on care homes and their management of residents with COVID‐19 before, after and during easing of lockdown. Aim to develop guidance on how care homes react in future outbreaks.	NA	
Martin AH/V012770/1 (UKRI)	5	UK	NR	NR	NR	Care homes	Determine how best to ensure that the human rights of residents of locked‐down care homes are protected, during lockdown and beyond.	NA	
Fotaki ES/V015338/1 (UKRI)	5	UK	NR	NR	NR	Care homes	Assess the financial impact of COVID on the UK care home sector	NA	
Surr 2021^102^ (NIHR)	5	UK	Mixed methods	NR	NR	Care homes	Explore current visiting practices for relatives of care home residents with dementia across England, best practice approaches and barriers and facilitators to these.	NA	
Fitzpatrick 2021^103^	5	UK	Mixed methods	In depth study of 6 care homes.	NR	Care homes	Investigate the challenges and solutions to protecting older people living in care homes from COVID.	NA	
Tischler AH/V006991/1 (UKRI)	5	UK	NR	NR	NR	Care homes	Develop creative activities to support health and wellbeing and alleviate social isolation and loneliness for people with dementia in care homes.	NA	
Vaitheswaran 2020^104^	1	India	Qualitative semi‐structured interview	31 dyads	AD, FTD, DLB, VD, mixed AD + VD, PD	Home‐dwelling	To describe the experiences and needs of caregivers of dementia during COVID‐19 pandemic.	Negative impact of restrictions on patients and carers, who expressed a need for more services and support during and after the pandemic. Video and telephone consultations can reach some, but lack of access to or knowledge of how to use technology were limitations. Technology should be used when feasible but in‐person services should continue with PPE and social distancing.	Access to and use of remote support and communication
Sorbara 2020^105^	1	Argentina	Cross‐sectional survey	324	MCI, AD, PD, FTD, DLB, VD	Home‐dwelling	Study of the different consultation modalities during quarantine.	Most patients experienced behaviour changes and over half of carers reported increased care burden. Only half accessed a medical consultation. Most participants >60 years preferred the telephone, only 9% used video and they were <60 years.	
Monin 2020^106^	1	USA	Cross‐sectional survey	161 (86) dyads	AD, VD, FTD, other	LTC facilities	Assess which methods of communication were associated with positive or negative emotional experiences for LTC residents and their families and friends.	Phone and email between family and friends were associated with more positive emotions.	
Zamir 2020^107^	1	UK	Qualitative	22 (7)	NR	3 care homes	Video calls to improve socialization between older adults and their peers	Video calls can reduce loneliness in residents and are feasible and acceptable.	
Lai 2020^108^	1	Hong Kong	Prospective cohort	60 dyads	Neurocognitive disorders in older adults	Home‐dwelling	Wellbeing measures after video conference versus telephone only over 4 weeks.	Video conference was superior to telephone only, for patient and carer wellbeing.	
Hammondcare International Ltd (Innovate, UKRI)	5	UK	NR	NR	NR	Care home	Develop a dementia consultancy service that uses video conferencing to provide care home staff with 1‐to‐1 time with a dementia consultant.	NA	
Mantrah Ltd (Innovate, UKRI)	5	UK	NR	NR	NR	Home‐dwelling	Develop a virtual knowledge base and chatbox for caregivers for people with dementia to reduce stress and carer burden.	NA	
Windle et al., 2023^109^	5	UK	RCT	356 dyads	NR	Home‐dwelling	Effectiveness and feasibility of an e‐health intervention for reducing dementia carer stress.	NA	
Savla 2020^110^	1	USA	Cross sectional	53 dyads	NR	Home‐dwelling	Assess caregivers' appraisal of COVID‐related stressors, support availability and coping strategies as predictors of perceived role overload.	Caregivers who were more concerned with the pandemic or received insufficient support from family/friends were more likely to experience role overload, compared to those who recognized positive aspects of the pandemic.	Home care support
Giebel 2020^68^	1	UK	Qualitative	15	AD, VD, young‐onset	Home‐dwelling	Explore the decision‐making process of continued paid home care support during the pandemic.	Unpaid carers made difficult decisions about whether to continue paid home care, and those that discontinued, had to increase the care hours themselves.	
Giebel 2020^111^	1	UK	Qualitative	42 dyads and 8 patients	AD, VD, young‐onset	Home‐dwelling and care home	Explore the effects of COVID‐related social care and support changes on lives of carers and patients.	Carers and patients were concerned about when services would re‐open, and carers worried about whether the person they cared for would still be able to re‐join support services.	
Geyer 2020^112^	1	Germany	Prospective cohort	21 dyads	NR	Home‐dwelling	To describe the experiences of people with dementia and their carers at 2 points during the pandemic.	Caregivers and people with dementia feel stressed by the pandemic but often have coping strategies, especially informal support.	
Cohen 2020^113^	1	Argentina	Cross sectional survey	80 dyads	AD	Home‐dwelling	Study of how social isolation due to COVID impacted carer stress and burden.	COVID confinement increased carer stress, especially for those caring for people with severe dementia. Carers of people with severe dementia worried about the availability of paid home care.	
Egunsola 2020^114^	1, 3	USA	Rapid review	5 studies	Cognitive impairment including dementia	Home‐dwelling	Review the best practices for care and engagement of older people with cognitive impairment who are required to isolate due to COVID‐19.	Telehealth is important for well‐being, it is important for them to receive continuous cognitive and environmental stimulation, caregivers require social support. No recommendations in hospitals/LTC facilities identified.Two of the included studies were not relevant for this review; one was a commentary article and the second investigated intellectual disabilities.	
Lorenz‐Dant 2021^49^	2, 3	Various	Rapid review	40 studies	NR	Unpaid carers	The impact of COVID on unpaid carers and the measures to support them.	Unpaid carers in community reported changes in care responsibilities, concerns around COVID infections, changes in support availability, financial and physical/mental health implications. Unpaid carers of people in residential care settings reported difficulties in communicating with residents, concerns about quality of care and COVID‐19 entering the care home. We also found that technology, financial assistance and support for working carers can help to mitigate these effects.	
								See also Carers UK report.	
BanerjeeES/V005529/1 (UKRI)	5	UK	NR	266	NR	Home‐dwelling	DETERMIND‐C19 project. Impact of COVID on patients and carers and identify predictors of better or worse outcomes. Data will be used to generate practical guidance for services and families.	NA	
Banerjee ES/S010351/1 (UKRI)	5	UK	NR	NR	NR	NR	DETERMIND project. Gather empirical data on how to support patients with dementia and their carers and identify predictors of better or worse outcome to generate practical guidance for services and families.	NA	
Maiden et al., EP/P010024/1 (UKRI)	5	UK	NR	NR	PD and dementia	Home‐dwelling	Develop and evaluate a computerized toolkit for patients and carers to promote self management of their condition.	NA	
Dowsett et al., 2020^115^	3	Canada	Registered systematic review	NA	NA	Home‐dwelling or supported living facility.	Rapid review of best practices for isolation and quarantine due to COVID‐19 for individuals with cognitive impairment.	NA	
Rare Dementia Support (RDS) Impact study^116^	5	UK	NR	NR	Rare, atypical or young‐onset dementia	Home‐dwelling	Study capturing the support needs of people living with or alongside a rare dementia, and the timepoints at which parts of the support service are typically requested (i.e. in both ‘typical' circumstances or in specific contexts, e.g. during the ongoing coronavirus pandemic).	NA	
Clare ES/V004964/1 (UKRI)	5	UK	Survey and interview	300 (50) dyads	NR	Home‐dwelling	Assess the impact of COVID pandemic on people with dementia and their carers and use the data to develop a set of resources to support them.	NA	
Giebel 2020^117^	1	UK	Survey	569 (61)	AD, mixed, vascular, other	Home‐dwelling	Impact of COVID‐related social support closures on older adults, dementia patients, and carers' well‐being, Apr‐May 2020.	Reduced social support and access during COVID‐19 pandemic, which was related to poorer wellbeing in all groups.	Access to and use of social care.
Berridge 2020^118^	6	USA	Interviews and surveys	45 senior leaders of health and social care in Washington State	NR	NR	Washington State health and social care services support 1.7 million older adults including over 107,000 with dementia. Report examines the impact of pandemic on health and social care usage from provider perspectives.	Identifies challenges confronting service delivery and care and findings relevant to policy and practice, such as the risk of delayed access to health care, exacerbation of problems.	
Samsi et al., 2021 (funded by Alzheimer's Society)^119^	5	UK	Interviews	NR	NR	Home‐dwelling	Investigate the use of residential respite services for people with dementia 2019‐2021. Adjustments made to include evidence of COVID impact.	NA	
STRiDE project^120^	6	Seven middle income countries	Report	NA	NR	NR	Adjustments made to the Strengthening responses to dementia in developing countries (STRiDE) project to include evidence on COVID impact and responses.	NA	Resources to support the national response to COVID‐19.
Vedel 2021^121^	6	Canada	Mixed methods				Study on impact of pandemic and health and social needs of people with dementia to make evidence‐based recommendations to improve policy.	NA	
LTCcovid project UK report^18^	6	UK	Report	NA	NR	NR	Gathered resources on the impact of COVID on dementia and resources for support.	Dementia care has been impacted on many different levels in the UK. Concerning practices were identified, such as lack of testing and PPE in care homes or mass signing of DNAR forms. Effects of prolonged lockdown or post‐COVID hospital discharge need further investigation.	
LTCcovid project cross‐country report^3^	6	UK, Spain, Ireland, Italy, Australia, USA, India, Kenya, Brazil	Report	NA	NR	NR	Gathered resources on the impact and mortality of COVID in dementia.	People with dementia accounted for 25% of all COVID‐19 related deaths in England and Wales, 31% in Scotland and 19% in Italy, which may be linked to high death rates in care homes. Rights to access treatments, e.g. intensive care, may have been compromised. Initiatives to allow visits to care homes and access healthcare are needed.	

*Note:* Type: 1 = published article, 2 = preprint, 3 = review, 4 = study protocol, 5 = (funded) study to be completed, 6 = national or international report.

Abbreviations: AD, Alzheimer's disease; DLB, Lewy body dementia; FTD, frontotemporal dementia; LTC, long term care; MCI, mild cognitive impairment; MDT, multidisciplinary; NA, not applicable; NR, not reported; PD, Parkinson's disease; PPE, personal protective equipment.

**TABLE 6:** Living well

Study	Type	Country	Type of study	Number of participants (with dementia if applicable)	Type of dementia	Setting	Focus of study	Findings	Themes
Quinn 2020^122^	1	USA	Prospective cohort	27	PD	Home‐dwelling	Fast recruitment to a rapidly modified physical activity coaching program in response to COVID‐19.	Telehealth may be feasible for physical activity coaching programs.	Remote monitoring and interventions.
Alves 2020^123^	1, 3	NA	Systematic review	NA	AD, VD, PD	Home‐dwelling	Psychoeducational and psychosocial measures that can reduce neuropsychiatric symptoms and carer burden at home. Included studies between Jan 2010‐Apr 2020.	There is evidence that increased use of technology can potentially benefit patients and carers during COVID pandemic.	
Fernandez‐Ruiz 2020^124^	1	Spain	Prospective	33	Half had neurodegenerative disease	NR	Determine the effectiveness of a telehealth consultation to evaluate nutritional status and quality of life in older people.	Telehealth is a viable approach to assess quality of life in relation to nutrition.	
Ballard MR/V027794/1 (UKRI)	5	UK	Clinical trial	NR	NR	Care homes	A digital version of a person‐centreed care and psychosocial intervention (WHELD), adapted for the COVID‐pandemic.	NA	
Manchester Camerata Ltd (Innovate, UKRI)	5	UK	NR	NR	NR	NR	Develop an online platform to deliver a music therapy programme to people with dementia during COVID.	NA	
Cousins 2020 ^125^	1	UK	Qualitative media analysis review	NA	NR	Care homes	Understand the extent to which ethical care was delivered to people with dementia in care homes.	There was a mixed picture of ethical care for residents. There were examples of selfless good practice, as well as examples of where ethical care has not been achieved.	Ethical care
Care Quality Commission (CQC)^126^	6	UK	National inquiry	NA	NR	Care homes	Report to understand how advance care decisions, including do not attempt resuscitation orders, were applied to groups of people during the pandemic, including those with dementia in care homes.	CQC received feedback from stakeholders, people who use services and their families and carers, that ‘blanket' DNACPR decisions had been proposed at a local level in a number of cases. People with dementia felt they were not supported to the extent they needed to be in advance care planning conversations, or given information they needed in an accessible way.	

*Note:* Type: 1 = published article, 2 = preprint, 3 = review, 4 = study protocol, 5 = (funded) study to be completed, 6 = national or international report.

Abbreviations: AD, Alzheimer's disease; DLB, Lewy body dementia; FTD, frontotemporal dementia; LTC, long term care; MCI, mild cognitive impairment; MDT, multidisciplinary; NA, not applicable; NR, not reported; PD, Parkinson's disease; PPE, personal protective equipment.

**TABLE 7:** Dying well

Study	Type	Country	Type of study	Number of participants (with dementia if applicable)	Type of dementia	Setting	Focus of study	Findings	Themes
Bolt 2020^127^	1, 3	Netherlands	Rapid scoping review	NA	NR	LTC facilities.	Palliative care and COVID‐19 in older adults and older patients with dementia to inform nursing recommendations.	There were no primary research studies on palliative care and COVID‐19. Recommendations from several articles were collated.	Palliative care
West 2020^128^	1, 3	NA	Rapid review	NA	NA	NA	Review of decision making for place of care and death in older people and apply learning to COVID. Studies up to Apr 2020 were included.	The decision‐making process for older people is affected by many factors, which can influence their and their caregivers' experience of illness and dying. Within the context of COVID, such decisions may have to be made rapidly and respond to changing needs.	Advance care planning and decision making.
Brazil et al., ES/V004255/1 (UKRI)	5	UK	Prospective cohort	99 family cares and nursing home staff	NR	Nursing homes	Develop and evaluate an online advance care planning COVID‐centric intervention for nursing homes, to improve end of care life.	NA	
Davies et al. ES/V003720/1 (UKRI)	5	UK	Mixed methods	NR	NR	NR	Develop an evidence‐based decision tool for family carers and people with dementia to use when making difficult decisions.	NA	

Preventing Well: Keeping fit and active to prevent the mental and physical consequences of isolation (Table 2).

The overall negative impact of COVID‐19‐related restrictions on people living with dementia and their carers' wellbeing, mental health and functioning was explored by over 30 publications, which included participants with a range of different dementias.^129^ A number of studies included in the Supporting Well and Living Well domains, described below, have the potential to help prevent the negative impact of further waves of the pandemic on dementia wellbeing. Most of the dementia‐focused studies used cross‐sectional surveys and relied on subjective retrospective ratings, and few compared findings with pre‐pandemic outcome data, revealing a need for further quantitative and longitudinal research.

Most studies were also based on community‐dwelling dyads, and more research is needed on the impact of restrictions on the wellbeing of care home residents with dementia, as well as care home staff and other practitioners who work with people living with dementia. Future data on these aspects in the UK may emerge, especially if findings specific to people affected by dementia are analysed and reported within broader studies. A number of such ongoing or completed broader studies in care home residents and staff, the health and social care workforce, and individuals and families were identified by the expert group and are listed in Table 2 and described in more detail in Table S2.

Another theme, related to the prevention of COVID‐19 in dementia, was the reduced ability of many people living with dementia to understand and comply with pandemic restrictions, social distancing and the use of face masks,^50^, ^51^, ^52^, ^53^ potentially increasing vulnerability to COVID‐19 infection. Long‐term care facility characteristics, such as size, degree of crowding, low levels of testing and personal protective equipment (PPE) were related to more COVID‐19 outbreaks.^58^, ^59^, ^60^ Available published COVID‐19 vaccine trial data indicate that all participants provided written consent (implying preserved mental capacity) and none had dementia.^130^ It will be important to monitor the effects of vaccines in people with dementia, many of whom will likely lack mental capacity to consent to vaccination and usually have multiple physical co‐morbidities.

Published studies also explored risk factors for more severe COVID‐19 infection in people with dementia, such as APOE4 homozygosity,^54^ institutionalization (residence in long‐term care facilities) and physical comorbidities.^9^ ^,^ ^56^, ^57^, ^64^

The consensus group felt that health and socio‐economic inequalities were important and under‐researched in many dementia studies, and future studies should ensure that research on COVID‐19 is inclusive,^131^ encompassing people with a range of socio‐demographic characteristics such as ethnicities. The DETERMIND‐C19 study (Table 5, Banerjee et al., UKRI) is investigating inequalities and inequities in dementia care and outcomes related to the COVID‐19 pandemic. Health inequalities are also present in other countries and vary between countries, which requires further investigation and policy solutions.^132^

*Diagnosing Well*: *Maintaining diagnostic services and awareness of symptoms of COVID‐19* ( 3).

Reduced access or referral to diagnostic services and reduced diagnosis rates related to the pandemic were described.^4^ This was despite the reported increased use of telemedicine and in contrast to overall increased mental health secondary care referrals.^78^ In England, the national dementia diagnosis rate has declined steadily since February 2020, dropping to 62.7% in November,^133^ lower than the national target of 66.7%^134^ and the previous year (68.2% in November 2019). Future research is needed to understand the impact of the loss of face‐to‐face assessments and access to diagnostic services during the pandemic.

Alternative methods to communicating with people affected by dementia (e.g., via telephone or video calls) have arisen in response to the disruption of normal service provision.^76^ The feasibility of remote assessment and virtual diagnosis of dementia in home‐dwelling participants during the COVID pandemic was studied,^70^ ^,^ ^135^; and members of the group were aware that the UK Dementia Research Institute (DRI) Care Research and Technology Centre (CR&T) is working on developing digital tools for remote cognitive testing to support memory clinics and a remote technology programme to detect signs of COVID‐19 infection in people with dementia at home (Sharp and Barnaghi, UKRI). Studies or reports that reviewed options for remote cognitive assessments or virtual memory clinics during the pandemic^72^ ^,^ ^73^ ^,^ ^74^ ^,^ ^75^ ^,^ ^76^ cautioned that reliance on technology risks excluding sections of the population, for example, those who cannot use or possess the technology, and could thereby worsen pre‐existing inequalities.

Eight studies reported that presentation of COVID‐19 in dementia is often asymptomatic or atypical and may include hypoactive delirium, making it more difficult to detect without accurate polymerase chain reaction (PCR) tests, especially as self‐reporting of symptoms can also be unreliable (Table 3). Despite this vulnerability among people living with dementia, a London‐based study showed that testing was only available at a later stage in older adults' psychiatric wards versus general hospitals at the start of the pandemic during the first UK lockdown, which was associated with outbreaks.^67^ A London‐based study of four nursing homes, where most residents (57%) had dementia, reported that 40% of COVID test‐positive residents were asymptomatic and there was a mortality rate of 26%,^68^ highlighting the need to prioritise nursing home testing and rapid infection control measures in this setting. Replication of studies conducted during the start of the pandemic at later periods, for example, subsequent lockdowns, would establish whether infection control improved and if high rates of SARS‐CoV‐2 antibody seropositivity^68^ had any impact on infection and mortality rates.

No studies had yet investigated the prevalence of post‐COVID syndrome, or ‘Long COVID’^136^ ^,^ ^137^ in people with dementia, which has the potential to worsen functioning and cognitive impairment.^138^ The potential impact of post‐COVID syndrome on the dementia workforce and carers is also unknown. Members of the expert group were aware of NIHR and UKRI‐funded studies on the longer term biological and health impacts of COVID‐19, that is, post‐COVID syndrome, to be announced in early 2021,^139^ the findings of which may be relevant to people affected by dementia. Advocating for people living with dementia to participate in ongoing research projects that are monitoring post‐COVID syndrome will be important, as will the inclusion of long‐term care facilities as sites for recruitment.

As COVID‐19 may increase dementia risk directly or by reducing cognitive reserve,^140^ it would also be important to review the biomarker evidence of progressive neurodegeneration or neuroinflammation in dementia following COVID‐19 infection. Fluid biomarker studies in people with dementia during the COVID‐19 pandemic would require implementation of procedures to minimise the risks to participants, staff and researchers.^141^

Treating Well: Ensuring access to the best treatment available (Table 4).

Primary care services provide healthcare for people affected by dementia in the community, but one UK survey found that most care home managers reported that general practitioners (GPs) were reluctant to visit care homes.^4^ In November 2020, the proportion of people living with dementia who received a medication review organised by their GP in the past 12 months decreased to 17.5%^133^ from 20.7% in November 2019.^134^ The longer‐term impact of these figures on the mental and physical health of people affected by dementia needs further investigation.

Teleconsultation used in memory clinics during lockdown^80^ ^,^ ^81^ ^,^ ^87^ was helpful for some carers, but others were uncertain about their purpose and usefulness.^5^ An adapted UK study (PriDem) found that many people living with dementia and their family carers were avoiding contact with primary health services and had mixed opinions of the value of telephone contact from primary care.^88^

Worsening of dementia‐related behaviours seen during the pandemic, such as anxiety, agitation and psychosis, was associated with an increase in antipsychotic prescribing.^95^ ^,^ ^94^ ^,^ ^96^ Remote management, for example, via video and telephone, was possible and helpful for crisis and multidisciplinary team reviews, but only a small proportion of participants in these studies had dementia.^86^ ^,^ ^135^ More research is needed to explore how remote support can be optimised in health and social care services and assess the success of any transitions to remote technologies. Several studies are investigating remote delivery of psychosocial or physical interventions to help to improve wellbeing and behaviour (see ‘Living well' domain below).

Reductions in access to services and the negative impact of the pandemic on dementia‐related behaviour may have contributed to a rise in dementia‐related emergency admissions amidst an overall decrease in psychiatric admissions to hospital.^93^ Patients with dementia had particular needs in hospital, as reported by a UK study,^91^ and required high levels of medical, nursing and dietician input and early discussions relating to end of life care and resuscitation status. Although older adult psychiatric/mental health wards in London introduced swift adaptations such as isolation, testing and oxygen treatment, these efforts were hampered by delayed access to Personal Protective Equipment (PPE) and COVID‐19 tests (compared to local general hospitals).^67^ There have also been inappropriate ‘blanket' do not attempt resuscitation (DNAR) decisions and restricted access to healthcare affecting care homes in England, which led to national investigation by the regulator.^126^ Similar concerns about unequal access to treatments were echoed in a US study.^65^ These measures are not supported by findings from one study in China, which found that hospitalised patients with dementia had better outcomes than patients without dementia, related to earlier hospital admission and care.^92^

Four centres^80^ ^,^ ^82^ ^,^ ^85^ ^,^ ^83^ published their experiences of the impact of the pandemic on clinical research activities. There was a reduction in patient recruitment and in‐person visits, but participation rates in ongoing studies remained high. Centres adapted to the pandemic by introducing COVID‐19 symptom monitoring, PCR testing of staff and participants, PPE use and/or teleconsultation. The longer‐term impact of COVID‐19 on research recruitment and participation, including initiatives such as NIHR Join Dementia Research (www.joindementiaresearch.nihr.ac.uk/) needs further investigation. It will also be important to obtain data on the impact on dementia researchers, especially early career researchers who may have lost opportunities for funding and support, and have experienced changes to academic workplaces, such as laboratories and universities.

Supporting Well: Providing personalised care and support to carers at home and people in care homes (Table 5).

At least six funded studies in care homes are ongoing (Table 5) to further understand the impact of COVID‐19 in UK care homes and develop future guidance and best practice approaches on visiting, protection of human rights, management of outbreaks, reducing social isolation and antimicrobial prescribing. Use of email, video and phone calls to contact family members and other care home residents may help reduce loneliness and improve wellbeing^106^ ^,^ ^107^ and remote input from healthcare professionals may help to support care home staff (HammondCare International Ltd, UKRI). Further research is needed to assess the extent to which all care home residents can access the potential benefits of remote support and communication.

The pandemic is also having a negative impact on the mental and physical wellbeing of family carers (see Preventing Well, Table 2), many of whom are unpaid and have experienced changes in care responsibilities (increased care hours) with financial implications, suggesting that financial assistance is a potential source of support.^49^ Studies and reports of home care services found that increased levels of carer stress could be mitigated by visiting care workers^112^ ^,^ ^110^ although family carers were often concerned about staff availability and potential risk of infection, so sometimes did not continue with such services.^142^ ^,^ ^113^ ^,^ ^17^ Other family carers also worried whether home care services would be available in future if they refused them,^142^ especially as they feared reductions in support and missed the continuity of a familiar home care worker. Six funded UK studies will investigate the impact of COVID‐19 on dementia care at home to identify predictors of better or worse outcomes, identify best practices and develop guidance, and one study (part of the Alzheimer's Society PriDem project) is investigating the role of family carers in managing risks of harm and of loss of autonomy.^143^

Studies that explored the use of remote communication technologies at home found that a substantial proportion of people living with dementia were unable to access remote consultations, possibly related to lack of access, knowledge or confidence to use the technology.^104^ ^,^ ^105^ People's preferences also varied.^105^ ^,^ ^108^ A partnership between UK DRI CR&T, the Alzheimer's Society and University of Worcester has built a digital resource to help groups and individuals in the UK who support people with dementia set up local online networks to replace the pre‐COVID face‐to‐face meetings (www.communitymakers.co), and two UK funded studies (Mantrah Ltd, UKRI^109^ NIHR) are developing digital or online interventions for families caring for someone living with dementia to reduce carer stress.

Only one published study reported findings relating to reduced access to and use of day services and respite care during the pandemic,^111^ although a number of studies relating to social care in the UK are planned or ongoing, such as a UK study that started before the pandemic on the use and experiences of residential respite services for people living with dementia and has continued during the pandemic.^119^ The ability of respite and daycare services to survive and ‘bounce back’ is important to understand as these services provide vital support for people with dementia and their carers. Several ongoing and completed broader UK studies on the impact of COVID‐19 on social care services and the social care workforce are summarised in more detail in Table S2.

Lastly, several dementia‐related resources and projects have been set up or adapted to support the national responses to COVID‐19 in the UK,^117^ Canada,^121^ and internationally^3^ ^,^ ^10^; including middle‐income countries.^120^

Living Well: Optimising the lived experience of dementia (Table 6).

Published and ongoing studies on the potential of technology to deliver physical, psychoeducational and psychosocial interventions remotely to people with dementia;^122^ Alves et al., 2020^124^ (Ballard; Manchester Camerata Ltd, NIHR) will offer insight into the acceptability of telehealth for those with more severe cognitive problems. Further research on the remote delivery of social care or befriending initiatives would also be important. The ethics of care provided to people with dementia in care homes were considered in two publications (Care Quality Commission^126^ ^,^ ^125^ Studies addressing the impacts of reduced social care services on people with dementia have been noted in the previous domain ‘Supporting well’.

Ongoing studies are assessing physical activity^48^ and deconditioning in dementia during the pandemic,^84^ and it would be important for future research to investigate the rehabilitation needs of people affected by dementia who are diagnosed with post‐COVID syndrome (Long Covid) or experience an exacerbation of other health problems as a result of isolation or reduced access to health and care services.

Dying Well: Ensuring the needs of people with dementia are met at the end of life (Table 7).

We found one published review^127^ but there were no primary research studies on palliative or end of life care relating to COVID‐19 and people living with dementia. Studies of palliative care services are underway and are likely to include people living with dementia. The decision‐making process in older adults may need to happen more rapidly in the context of COVID‐19,^128^ and two ongoing UK studies are developing advance care planning and decision‐making tools for people affected by dementia in care homes with nursing (Brazil et al., UKRI, Table 7) and family carers.^144^ UKRI, Table 7). The latter study has produced a freely available evidence‐based decision tool to help people with dementia and their family carers make difficult decisions.^144^ Such decisions and consultations are likely to be hampered by the loss of visitor access to many hospitals and care homes during the pandemic, but quantitative research in this area is lacking.

Additional studies as well as the regulator's investigations^126^ on the use of advance decisions to refuse treatments and misuse of DNAR orders may help improve advance care planning for people living with dementia.

**Box 1:** Summary of findings and consensus directions for future research

Findings from previous/current research	Directions for future research
Preventing well:	
Negative impact of COVID pandemic on cognitive, mental and physical health in people with dementia and their carers. Many family/friend carers provided more care, with financial implications. Impact of COVID on the general health and social care workforce. Prevention of COVID‐19 infection: Many people living with dementia had a reduced ability to understand and comply with pandemic restrictions and wearing face masks. LTC facility‐related factors were related to COVID‐19 outbreaks. COVID‐19 risk factors in people with dementia included APOE4 homozygosity. Living in a care home and comorbidities increases risk of COVID‐19 disease severity.	Quantitative and longitudinal studies on the impact of COVID‐19 and isolation. Studies should be inclusive and assess the impacts of inequalities. Impact of restrictions on the wellbeing of care home residents with dementia, care home staff and health and social care professionals who work alongside people with dementia. Findings specific to people working with or affected by dementia within broader studies, and dementia‐specific findings within care homes with residents who have/do not have dementia should be analysed. Monitoring effects of vaccination in people with dementia, as they were not included in published vaccine trials. Longitudinal data on the impact of COVID‐19 on dementia risk.
Diagnosing well:	
Reduced access to dementia diagnostic services and reduced diagnosis rates during the pandemic. COVID‐19 in dementia is often asymptomatic or atypical and may include hypoactive delirium, making accurate PCR testing critical to control spread. At the start of the pandemic in some areas, people with dementia in care homes and psychiatric/mental health hospitals were only able to access COVID‐19 testing at a later stage compared to others. This likely contributed to local outbreaks. Remote assessment and virtual diagnosis of dementia in home‐dwelling participants during the pandemic may be feasible.	Impact of loss of face‐to‐face assessments and access to diagnostic services during the pandemic on dementia diagnoses and mental health diagnoses in people with dementia. Replication of studies conducted during the start of the pandemic at later periods, e.g., subsequent lockdowns, would establish whether lessons were learned and if they (and/or widespread antibody seropositivity) had any impact on infection and mortality rates. Optimise remote assessments and ensure those who cannot use or possess the technology are not left behind. Studies on post‐COVID syndrome (‘Long COVID’) to include people with dementia to investigate the prevalence and impact of this condition on dementia risk, diagnosis and progression. The potential impact of post‐COVID syndrome on carers and the dementia workforce. Longitudinal biomarker studies (imaging, fluid, cognitive) to investigate rates of dementia progression following COVID‐19.
Treating well:	
Reduced access to and provision of primary care and memory clinic services, alongside an increase in antipsychotic prescribing. Responses to telehealth were mixed. People with dementia who are hospitalised have specialised needs, but reduced access to health care, which may not be justified. Clinical research activities, such as participant recruitment, have been affected.	Longer‐term impact of reduced access to primary and secondary health services. The acceptability and effectiveness of telehealth for people living with dementia. Factors associated with positive treatment outcomes in people with dementia to inform inappropriate restriction to healthcare and use of DNAR orders. Impact of COVID‐19 on dementia research, including on existing and future projects, early career researchers, and participant recruitment initiatives.
Supporting well:	
The pandemic has reduced care home residents' and staff wellbeing. Telehealth may benefit some residents. Informal or paid home care, day care and other services can mitigate carer stress, but many carers worried about their availability and infection risk and did not access services. Many carers were unable to access remote consultations or support. Financial assistance may be beneficial. Reduced access to and use of social care services, e.g. respite care. Resources and projects set up or adapted to support national responses to COVID‐19.	Best practice in care homes, relating to visiting, remote communication, infection control whilst reducing loneliness and protecting human rights. How to best support dementia care at home, including social, digital, financial and other interventions to reduce carer stress and burden. Social care research findings related to older people in particular may be extrapolated to dementia, and specific dementia‐related findings should be analysed and reported within broader studies that include these groups.
Living well:	
Remote delivery of psychosocial, education and physical interventions have the potential to benefit wellbeing, but the caveats of access to and capacity to use technology also apply.	Ongoing and further research to assess the success of remote interventions to improve wellbeing. Rehabilitation needs of people affected by dementia, e.g., due to post‐COVID syndrome or prolonged isolation.
Dying well:	
Difficult decisions may need to be taken more rapidly in COVID‐19, and decision aids for people with dementia and their families are now available.	Primary research studies on palliative care involving people with dementia. Studies to help improve advance care planning.

## 4 DISCUSSION

Based on the findings that emerged from this initiative, there were directions for future research within each domain that the consensus group considered important (Box 1). Some themes applied across all domains, such as the potential benefits and risks of remote healthcare to deliver interventions and support, the need for longitudinal studies to monitor the longer‐term impacts, the risk and impact of widening health and other inequalities, the need for socio‐demographically inclusive studies, and the benefit of reporting dementia‐specific findings within broader studies.

As this is a fast‐moving and rapidly changing area in terms of policies and adaptations, which may outpace the rate of the normal publication process, we have tried to swiftly incorporate research findings from a range of non‐peer reviewed sources, including registered systematic reviews in progress, preprint databases and COVID‐19‐specific websites and reports. This paper presents findings from a literature review and expert consensus, intended to provide comprehensive coverage of several aspects of dementia research related to COVID‐19, and was not driven by a specific research question. Our study was not designed to be a traditional systematic or scoping review^145^ and we did not formally quantify the quality of studies. Therefore, our findings may be less comprehensive compared to a protocol‐driven search strategy that follows published guidelines and/or systematic critical appraisal.

Members of the consensus group are involved in online projects that can rapidly update evidence relating to COVID‐19, for example, the LTCcovid collaboration (www.ltccovid.org). While peer‐review remains the gold standard for research publication, databases such as these as well as preprint repositories can provide valuable, time‐critical resources during the pandemic.

Another limitation of our study was that some potentially relevant studies of long‐term care facilities may have been omitted if they did not explicitly mention inclusion of people with dementia: members of the consensus group identified some such studies, and these are described in the text. Non‐specific findings should be interpreted cautiously, including those from large population‐based studies/surveys of individuals, families and the health and social care workforce. For example, only 22% of respondents in the Carers UK survey (Table 2)^17^ were aged over 65 years (an unknown proportion were supporting family members with dementia), thus potentially limiting the generalisability of findings to dementia care.

Other limitations, related to the fast‐changing nature of the pandemic, were that the themes and research gaps identified in this study may not apply or become less relevant as the pandemic continues to unfold. At the time of writing, members of the expert consensus were aware that several projects have yet to be announced or have only recently been awarded funding, for example, the NIHR Policy Research Programme (PRP) announced further COVID‐19 related studies that may be relevant to dementia in early 2021. These future studies were not detailed in this paper but may fulfil additional themes within the Dementia Wellbeing Pathway or meet identified gaps in research. We structured our findings based on the NHS England Dementia Well Pathway, but studies were not conducted with this framework in mind. Thus, research findings may be relevant to more than one domain and the categorisation of studies, by necessity, may sometimes have been imprecise. As this study was prompted by a question from the English Dementia Programme Board on research on dementia and COVID‐19, and our consensus group were English‐based experts, our findings and consensus views were skewed towards research based in England.

Overall, the COVID‐19 pandemic has had a disproportionately negative impact on dementia wellbeing, and researchers and funding organisations have responded rapidly to try to understand the impacts. We have identified potential directions for future research to explore these further, so that evidence‐based measures can be developed to improve the quality of life of people affected by dementia.

## CONFLICT OF INTEREST

None.

## Supporting information

Supplementary Material

## Data Availability

Data sharing not applicable to this article as no additional datasets were generated or analysed during the current study.
