# Supplementary material for: Dementia wellbeing and COVID‐19: Review and expert consensus on current research and knowledge gaps
Source: Int J Geriatr Psychiatry. 2021 May 27;36(11):1597–639. doi: 10.1002/gps.5567 (PMC8237017; doi:10.1002/gps.5567)
Supplement: Supplementary file 1 — Supplementary Material [file GPS-36-1597-s001.docx]

Table 1: Background of consensus group members/co-authors:

| Name | Role/expertise | Institution |
| --- | --- | --- |
| Kathy Liu | MRC Clinical Research Training Fellow and Old Age Psychiatrist | University College London |
| Robert Howard | Professor of Old Age Psychiatry | University College London |
| Sube Banerjee | Professor of Dementia | University of Plymouth |
| Adelina Comas-Herrera | Assistant Professorial Research Fellow | Care Policy and Evaluation Centre, London School of Economics |
| Joanne Goddard | Senior Research Portfolio Manager | Economic and Social Research Council, UKRI |
| Martin Knapp | Professor of Health and Social Care | Department of Health Policy, London School of Economics |
| Gill Livingston | Professor of Old Age Psychiatry | University College London |
| Jill Manthorpe | Professor of Social Work | King’s College London |
| John O’Brien | Professor of Old Age Psychiatry and NIHR Clinical Research Network National Specialty Lead for Dementia. | University of Cambridge School of Clinical Medicine |
| Ross Paterson | NIHR Academic Clinical Lecturer in Neurology | University College London |
| Louise Robinson | Professor of Primary Care and Ageing | Newcastle University |
| Martin Rossor | Professor of Clinical Neurology & NIHR National Director for Dementia Research | University College London |
| James Rowe | Professor of Cognitive Neurology and Director of Cambridge Centre for Frontotemporal Dementia and Related Disorders. | University of Cambridge |
| David Sharp | Professor of Neurology and Director of Care Research and Technology Centre, UKRI | Imperial College London |
| Andrew Sommerlad | Principal Research Fellow and Consultant Old Age Psychiatrist | University College London |
| Aida Suárez-González | Research Associate and Clinical Neuropsychologist | University College London |
| Alistair Burns | Professor of Old Age Psychiatry and National Clinical Director for Dementia and Older People’s Mental Health at NHS England and NHS Improvement. | The University of Manchester |

Table 2: Further details on completed or ongoing broader studies that may be relevant for people affected by dementia.

| Dementia Wellbeing Domain | Further details on broader studies |
| --- | --- |
| Preventing Well | Members of the expert group were aware of completed or ongoing studies that could be relevant to people affected by dementia, especially if dementia-specific findings are reported. These included surveys or interview-based studies on the impact on individuals and families [(Understanding Covid, 2020)](https://paperpile.com/c/cKSBt2/jzyp0), health and social care workers [(Hussein et al., 2020; Woolham et al., 2020; McFadden et al., 2020)](https://paperpile.com/c/cKSBt2/JSDcY+vOPWN+X5u5n), the nursing workforce (ICON) [(RCN, 2020)](https://paperpile.com/c/cKSBt2/2KQtZ) and care home domestic staff (Samsi et al., in progress).  Specific UK care home studies additionally identified by the expert group, relevant to this domain, included a published small interview study reporting on the negative mental health of workers in care homes and domiciliary care agencies related to the pandemic [(Nyashanu et al., 2020)](https://paperpile.com/c/cKSBt2/s1WRx), a funded study investigating the challenges of social distancing and isolation experienced in care homes [(Fitzpatrick et al., 2021)](https://paperpile.com/c/cKSBt2/5b8Rc), and an ongoing NIHR Adult Social Care Research Unit (ASCRU) project monitoring rates of infection, deaths, staff absences and vacancies in care homes (Fernandez et al.). |
| Supporting Well | Members of the consensus group were aware of several ongoing and completed UK studies on the impact of COVID-19 on social care services and the social care workforce, the findings of which may affect people with dementia and their carers. These included an ongoing study on adult safeguarding, [(Keele University, 2020)](https://paperpile.com/c/cKSBt2/zrkqB), a published study on social care personal assistants (directly employed care workers) in England during COVID-19 [(Woolham et al., 2020)](https://paperpile.com/c/cKSBt2/vOPWN) and an ongoing inclusive study of their employers, who are likely to include people living with dementia or their carers (Samsi et al. 2021). Three studies are exploring changes to legal entitlements (easements) to local authority funded social care, which may affect people with dementia and their carers (Baginsky et al; McHale et al; Price et al.).  The impact of COVID-19 on the wellbeing of the health and social care workforce has been reported and was addressed earlier in the ‘Prevention’ domain, including a recently published UK first wave of a study of health and social care workers who work with older people, many of whom were likely to be living with dementia (McFadden et al., 2020) and will report on its two further waves in 2021. Although there was a published report on the impact of the pandemic on health and social care provision such as staffing reductions in Washington State, US (Berridge et al., 2020), members of the consensus group considered that research on workforce capacity and sufficiency have not been adequately addressed in the UK, especially within social care. As an exception, the findings from a large ongoing study of COVID-19 related experiences, morbidity and mortality among registered home care workers in Wales (Robling et al., UKRI) will include home care workers supporting people living with dementia by linking the workforce’s responses with their NHS data. |

REFERENCES

[Fitzpatrick J. et al. 2021. Protecting older people living in care homes from COVID-19: challenges and solutions to implementing social distancing and isolation.](Fitzpatrick%20J.%20et%20al.%202021.%20Protecting%20older%20people%20living%20in%20care%20homes%20from%20COVID-19:%20challenges%20and%20solutions%20to%20implementing%20social%20distancing%20and%20isolation.%20) [*NIHR Funding and Awards*. Available at:](http://paperpile.com/b/cKSBt2/5b8Rc) <https://www.fundingawards.nihr.ac.uk/award/NIHR132541> [(Accessed: 4 January 2021).](http://paperpile.com/b/cKSBt2/5b8Rc)

[Hussein S et al. 2020. COVID-19 and the wellbeing of the UK social care workforce. *Personal Social Services Research Unit*. Available at:](http://paperpile.com/b/cKSBt2/JSDcY) <https://www.pssru.ac.uk/resscw/files/2020/12/COVID19-and-the-UK-Care-Workers_FINAL_01dec20.pdf>[.](http://paperpile.com/b/cKSBt2/JSDcY)

[Keele University. 2020. Researchers to investigate impact of Covid-19 pandemic on adult social care and safeguarding. Available at:](http://paperpile.com/b/cKSBt2/zrkqB) <https://www.keele.ac.uk/research/researchnews/2020/november/impact-covid/adult-safeguarding.php> [(Accessed: 7 January 2021).](http://paperpile.com/b/cKSBt2/zrkqB)

[McFadden P, Gillen P, Moriarty J, Manthorpe J., *et al.* 2020. Health and social care workers’ quality of working life and coping while working. Available at:](http://paperpile.com/b/cKSBt2/X5u5n) <https://www.ulster.ac.uk/__data/assets/pdf_file/0006/681441/COVID-19-HSC-Workforce-Survey-May-July-2020.pdf> [(Accessed: 7 January 2021).](http://paperpile.com/b/cKSBt2/X5u5n)

[Nyashanu M, Pfende F, Ekpenyong MS. 2020. Triggers of mental health problems among frontline healthcare workers during the COVID-19 pandemic in private care homes and domiciliary care agencies: Lived experiences of care workers in the Midlands region, UK. *Health Soc Care Community*. DOI:](http://paperpile.com/b/cKSBt2/s1WRx)[10.1111/hsc.13204](http://dx.doi.org/10.1111/hsc.13204)[.](http://paperpile.com/b/cKSBt2/s1WRx)

[RCN. 2020. Researching the impact of COVID-19. *Royal College of Nursing*. Available at:](http://paperpile.com/b/cKSBt2/2KQtZ) <https://www.rcn.org.uk/magazines/bulletin/2020/june/icon-research-nursing-study-professor-kelly-covid-19> [(Accessed: 4 January 2021).](http://paperpile.com/b/cKSBt2/2KQtZ)

[Understanding Covid. 2020. Understanding Covid-19 survey. Available at:](http://paperpile.com/b/cKSBt2/jzyp0) <https://www.understandingsociety.ac.uk/topic/covid-19> [(Accessed: 6 January 2021).](http://paperpile.com/b/cKSBt2/jzyp0)

[Woolham J, Samsi K, Norrie C, Manthorpe J. 2020. The impact of the coronavirus (Covid-19) on people who work as social care Personal Assistants. NIHR Policy Research Unit in Health and Social Care Workforce, The Policy Institute, King’s College London DOI:](http://paperpile.com/b/cKSBt2/vOPWN)[10.18742/pub01-036](http://dx.doi.org/10.18742/pub01-036)[.](http://paperpile.com/b/cKSBt2/vOPWN)
